# Tibiofemoral axial rotation during the golf swing is influenced by total knee arthroplasty bearing type and foot rotation

**DOI:** 10.1002/jeo2.70529

**Published:** 2025-12-10

**Authors:** Renate List, Nils Horn, Samara Monn, Danielle Hinny, Philipp Bänteli, Kevin Wunderlin, Katja Oberhofer, William R. Taylor, Stephen J. Ferguson, Tomas Drobny, Stefan Preiss, Pascal Schütz

**Affiliations:** ^1^ Human Performance Lab Schulthess Clinic Zurich Switzerland; ^2^ Department of Hip and Knee Surgery Schulthess Clinic Zurich Switzerland; ^3^ Institute for Biomechanics ETH Zurich Zurich Switzerland

**Keywords:** implant design, injury risk, kinematics, stance, videofluoroscopy

## Abstract

**Purpose:**

Golf remains a popular sport after total knee arthroplasty, although many patients report mild pain during or after play, particularly in the lead knee (knee on the target side). Lead knee kinematics during the golf swing are characterised by high tibiofemoral axial rotation at low joint flexion. However, it remains unclear whether the same range of tibiofemoral axial rotation is observed with mobile versus fixed bearing designs. This study aimed to evaluate the influence of two implant designs (mobile‐bearing and fixed‐bearing) and lead foot rotation (self‐selected, 0° and 30° externally rotated) on implant kinematics during the golf swing.

**Methods:**

In a total of 11 healthy subjects (five mobile‐bearing and six fixed‐bearing), kinematic and kinetic data during the golf swing were assessed using videofluoroscopy, an opto‐electronic three‐dimensional (3D) motion capture system and two force plates.

**Results:**

Significantly greater ranges of tibiofemoral axial rotation in the lead knee during the golf swing were observed for the mobile‐bearing compared with the fixed‐bearing design, independent of foot rotation at the start. Furthermore, the present data showed that a change in foot rotation from a 0° to a 30° externally rotated lead foot increased tibiofemoral axial rotation of the mobile‐bearing design but not the fixed‐bearing design.

**Conclusion:**

The present results suggest that the mobile‐bearing design allows a greater range of tibiofemoral axial rotation in the lead knee than the fixed‐bearing design during the golf swing, likely due to the differences in internal transverse plane constraints between the two implant designs. However, it remains unclear whether the greater rotation is preferable in terms of longevity and soft tissue loading in total knee arthroplasty patients playing golf. The present data serve as input for musculoskeletal and finite element modelling with the future goal of guiding implant selection and foot positioning recommendations for golf after total knee arthroplasty.

**Level of Evidence:**

N/A.

**Abbreviations**
CR: cruciate‐retaining
FB: fixed bearing
MB: mobile bearing
ROM: range of motion
SD: standard deviation
SPM: statistical parametric mapping
TKA: total knee arthroplasty

## INTRODUCTION

Golf is a widely popular sport, especially amongst elderly people, due to its association with improved physical health, low impact and moderate risk of musculoskeletal injury [1, 44, 45]. Thus, the ability to play golf following total knee arthroplasty (TKA) is an often‐discussed topic that concerns a large population group: up to 20% of patients with TKA are reported to be golfers [52]. The members of the European Knee Associates recommend golf after TKA beyond 12 weeks [59], and the reported return‐to‐play rates of 80%–95% following TKA are extremely encouraging [21, 52, 61]. Nevertheless, knee joint injuries account for up to 18% of all injuries in golf [1, 4].

Based on biomechanical analysis, it has been decisively demonstrated that the golf swing produces a more stressful condition in the lead knee (knee on the target side) versus the trail knee (knee away from the target) [10, 14, 24, 25, 27, 43, 49, 58], which may contribute to accelerated polyethylene wear and could potentially explain the significantly higher pain levels reported by golfers with a TKA in the lead compared to the trail knee [38]. Insights from periodic X‐ray imaging in five healthy male golfers revealed more than 15° of rapid femoral axial rotation from the top of the backswing to the end of follow‐through at flexion angles <30° in the lead knee [43] and videofluoroscopy in one subject with a TKA in the lead knee revealed implant axial rotation of 13° at the top of the backswing to 2.7° at the end of follow‐through with simultaneous knee extension from 29.6° to 8.4° knee flexion [14]. For comparison, the range of internal–external rotation is almost double that presented for a cruciate‐retaining (CR) fixed bearing (FB) TKA during the stance phase of stair descent (7.1 ± 2.1°) [34], but is still clearly smaller than the maximal range of internal–external rotation assessed intraoperatively at 30° flexion when applying a physiologically allowable maximal rotation stress in a posterior stabilised TKA (21.5 ± 7.8°) [18]. Moreover, peak contact forces of up to 440% of body weight (BW) were previously measured in vivo in the lead knee during the golf swing by means of an instrumented TKA [42], which is approximately the force experienced when sitting down (220% BW) and 25% higher than when descending stairs (346%) [28].

Given the insights from biomechanical analyses, research has been conducted to provide postural recommendations for reducing the potentially harmful loading on the knee joint during golfing. Focus has thereby been given to the reduction of the peak external adduction moments through changes in stance width and/or foot rotation [19, 20, 27, 36, 37]. Indeed, postural changes to incorporate a wider stance width and/or increased toe‐out angle (lead foot external rotation) were previously shown to decrease peak external adduction moment of the lead knee following ball impact in healthy volunteers [19, 20, 37] and professional male golfers [27]. Based on these findings, it was suggested to teach an externally rotated lead foot to most ageing recreational golfers in order to decrease medial compartment loading and associated knee injury risks [37]. However, it remains unclear how changes in foot rotation may influence knee joint biomechanics in golfers with a TKA and whether different types of knee implants (i.e., FB vs. mobile bearing [MB]) may show different ranges of motion (ROMs) in the joint.

One of the theoretical advantages of MB versus FB TKA designs is the ability to axially rotate while maintaining congruence, thus reducing contact stress and wear of the polyethylene inlay, as well as potentially reducing shear forces at the bone–implant interface to avoid component loosening [12, 23, 46, 50, 62]. In contrast, the theoretical disadvantages of an MB design may be a higher loading on the surrounding soft tissue due to fewer kinematic constraints [2, 41, 60]. However, to date, large, randomised controlled trials have not revealed significant differences in clinical, functional and radiological outcome measures or patient satisfaction between MB versus FB designs at short‐, mid‐ or long‐term follow‐up [5, 16, 26, 63]. Only one meta‐analysis of 612 knees at 10 years of follow‐up showed a clear improvement in the Knee Society Function Score (KSFS) for MB versus FB designs, yet without a difference observed in complication rates between the two groups [17].

Given the specific motion pattern of the knee joint during golfing and its differences from regular daily activities, the question remains whether implant design matters in golfers after TKA. Based on existing literature, it is expected that the behaviour of a TKA in vivo is task dependent and most likely different between FB and MB designs [24, 50, 53, 55, 56, 65, 66]. However, inconsistent results have been reported regarding details of the differences, likely due to ongoing advances in prosthesis component designs and previous challenges of accurately assessing frontal and transverse plane kinematics during more demanding tasks in vivo. In early research, greater axial rotation and less condylar lift‐off were found in 20 patients with an MB compared with 20 patients with an FB TKA during weight‐bearing deep knee bending by means of dynamic videofluoroscopy [50], while no differences in posterior condylar translation and tibial axial rotation during weight‐bearing deep knee bending were later reported for the MB versus the FB implant design [56]. More recently, in a study investigating 29 subjects with an FB compared with nine subjects with an MB prosthesis, it was found that the MB design more closely reproduced axial rotation of the healthy knee during a lunge and gait with a pivot, suggesting similar results to be expected for activities with complex three‐dimesional (3D) kinematic patterns such as the golf swing [24].

In summary, golf‐specific lead knee biomechanics, characterised by a high tibiofemoral axial rotation at low joint flexion with a simultaneous high external knee adduction moment, have been associated with high joint compression and soft tissue loading [1, 6, 49], likely explaining some of the pain levels reported in patients with a TKA in the lead knee [38]. Yet, it remains unclear whether the same joint kinematics are observed in MB versus FB designs and whether changes in foot rotation influence TKA kinematics during the golf swing in vivo. Thus, the objective of the present study was to assess the influence of the bearing type as well as the lead foot rotation on TKA implant kinematics during the golf swing. It was hypothesised that tibiofemoral axial rotation is different between MB versus FB implant designs, as well as different for a 0° versus 30° rotated lead foot, despite similar external loading conditions measured during the golf swing in vivo.

## MATERIALS AND METHODS

### Subjects

A total of 11 healthy participants were recruited for this study. Participants were recruited through the Schulthess Klinik, Zurich, Switzerland, and all subjects provided written informed consent prior to study participation in accordance with local ethical regulations (BASEC‐Nr. 2018‐01763). The specific inclusion criteria were: right‐handed golfers with a unilateral TKA in the left lead knee (Persona CR FB TKA [Zimmer Biomet] or Attune^TM^ CR MB TKA [DePuy Synthes, Johnson & Johnson]), at least 2 years postoperative, a satisfactory clinical outcome with an Oxford Knee Score (OKS) [7] ≥ 40, playing golf at least once per week with a handicap between 0 and 30 and a body mass index (BMI) ≤ 33. Subjects were excluded if they had any significant problems in the lower limbs, pain during activities of daily living (visual analogue scale [VAS] > 2), misaligned TKA, signs of radiological lucencies or pes planovalgus ipsilateral.

### Data acquisition

**Figure 1:**
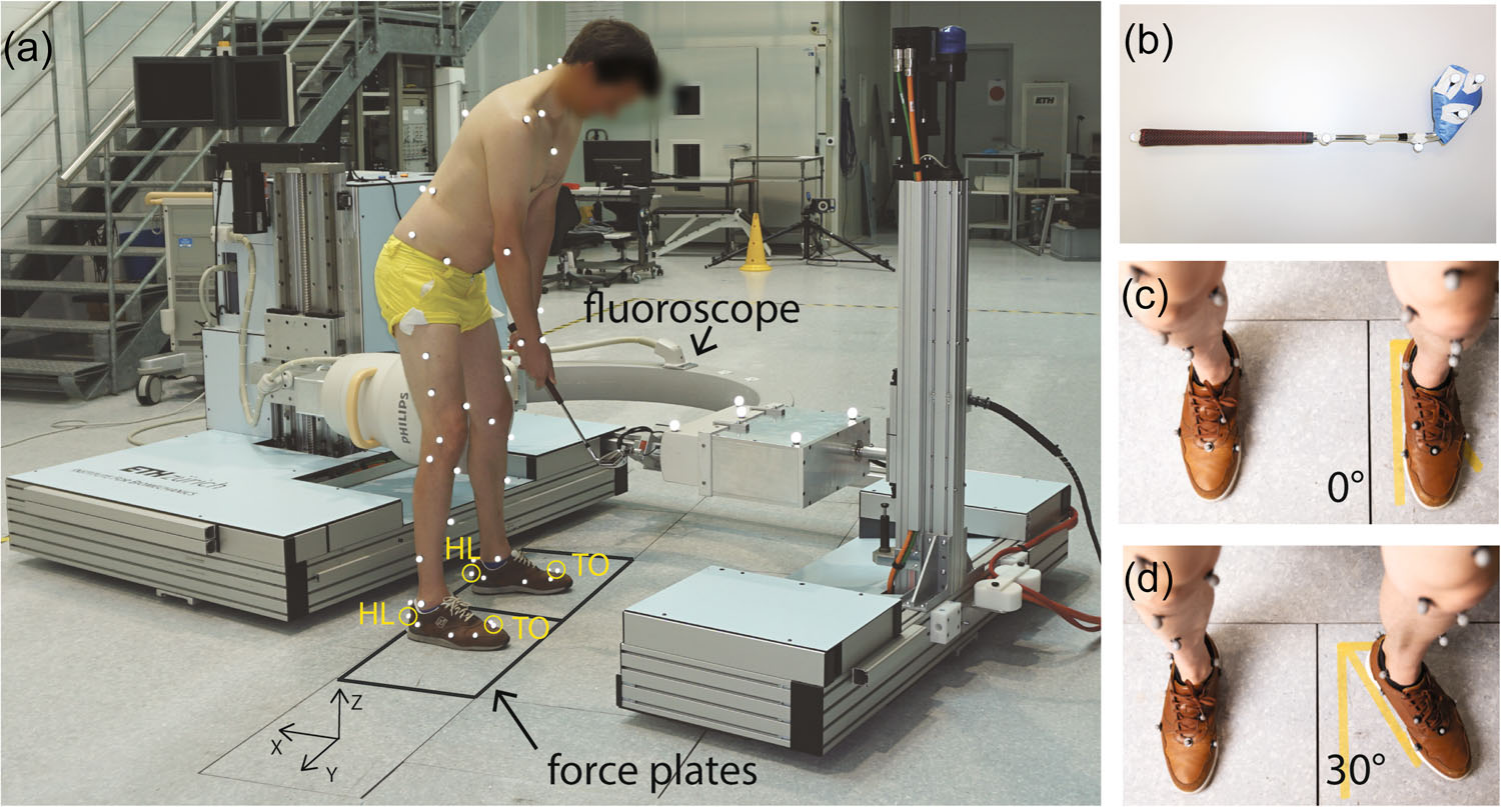
(a) Measurement setup including videofluoroscope, skin marker set for three‐dimensional (3D) optical motion capture [31], floor‐embedded force plates and modified golf club. In the present study, only the lower heel (HL) and second toe (TO) markers, as well as the markers on the club and the wrist, were used. All tibiofemoral kinematics were based on videofluoroscopy. The foot markers on the HL and the TO were specifically used to reflect the longitudinal direction of the foot. The lab coordinate system (*X*,*Y*,*Z*) is used to represent ground reaction forces. (b) The modified golf club had a length of 51 cm and additional weight at the club head. (c) and (d) Yellow tape marks were used to guide the subject's feet placement for the foot rotation with a 0° and 30° externally rotated lead foot.

Kinematic and kinetic data during the golf swing were assessed synchronously using a videofluoroscope [33], an opto‐electronic 3D motion capture system consisting of 22 infra‐red cameras (Vicon MX System, Oxford Metrics Group; sampling frequency 200 Hz) and two force plates (Kistler AG; sampling frequency 2000 Hz) that were fully decoupled from the surrounding floor (Figure 1a). To enable a full swing within the C‐arm of the videofluoroscope, a modified golf club was used (Figure 1b). The modified club was 51 cm in length and was equipped with additional weight at the clubhead to achieve similar characteristics in terms of overall weight and moment of inertia to an original seven‐iron club. After familiarisation trials using the modified golf club, kinematic and kinetic data of at least five full swing cycles were acquired within the videofluoroscope for two different controlled foot rotations and one self‐selected, preferred, uncontrolled foot rotation. The two controlled foot rotations were 0° (Figure 1c) and 30° (Figure 1d) externally rotated lead foot angles. Additionally, kinematic and kinetic data were assessed during an upright standing trial with legs straight and BW evenly distributed across both legs. Technical details of the videofluoroscope have been published previously [33], whereby fluoroscopic images of the TKA were acquired with a frequency of 30 Hz, a shutter time of 1 ms and an image resolution of 1000 × 1000 pixels.

### Data processing

**Figure 2:**
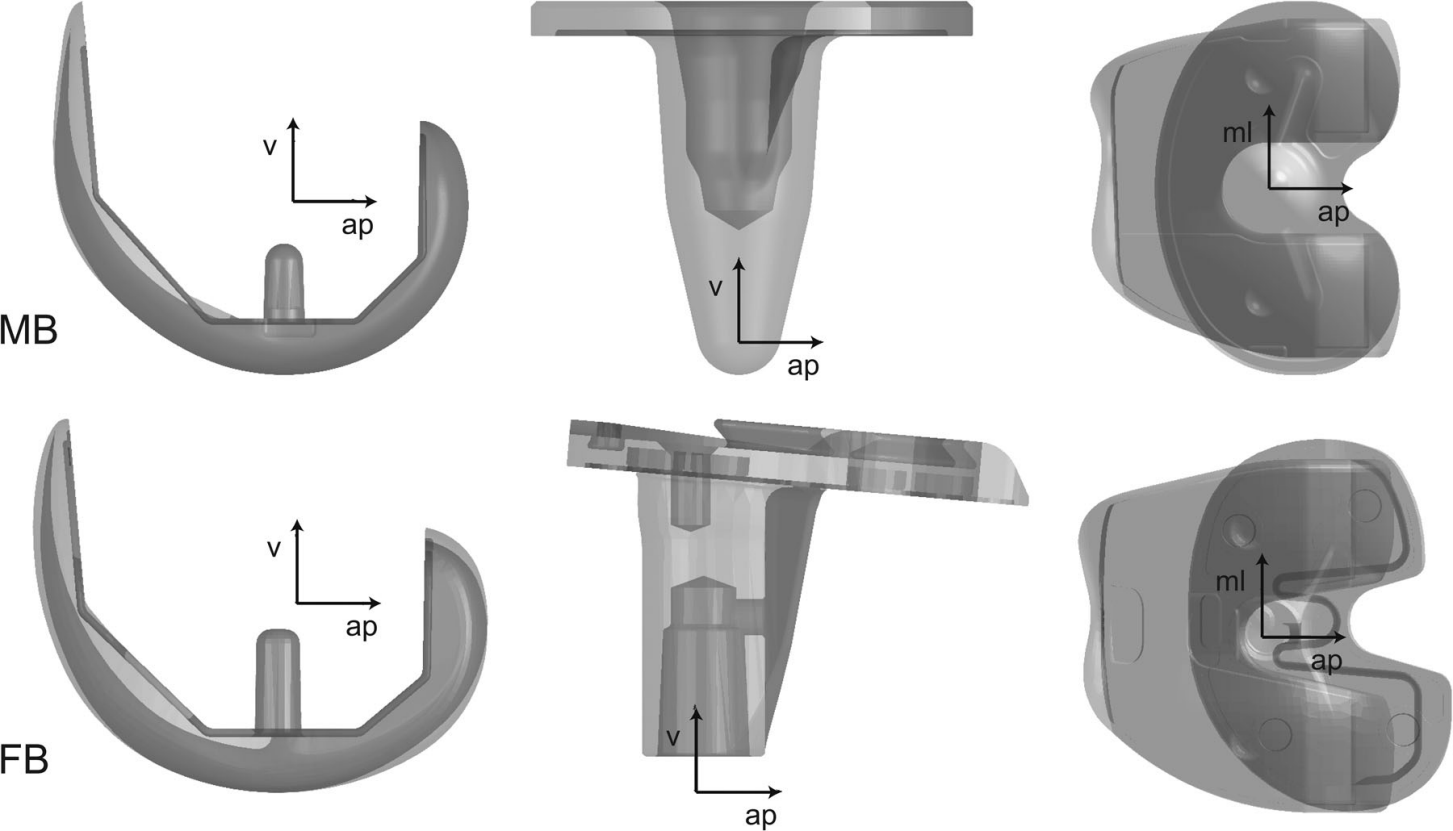
Implant coordinate system (black arrows) for the femoral and tibial components of the mobile bearing (MB) and fixed bearing (FB) total knee arthroplasty (TKA)—mediolateral (ml) axis, anteroposterior (ap) axis and vertical (*v*) axis.

Fluoroscopic images were distortion corrected based on a reference grid [11, 30]. Projection parameters of the fluoroscopic system (i.e., focal distance, location of the principal point in the image plane) were determined by a least‐squares optimisation method using five images of a calibration tube [11]. The 3D poses of the TKA components were determined by a two‐dimensional (2D)/3D registration using available computer‐aided design (CAD) models of the implant components. The registration algorithm was based on the approach developed by Burckhardt et al. [3], with reported registration errors of ≤ 0.25° for all rotations, 0.3 mm for in‐plane and 1.0 mm for out‐of‐plane translations for a similar TKA [11]. The relative rotations between the femoral and tibial components of the MB and FB designs were determined based on the corresponding femoral and tibial implant coordinate systems (Figure 2) using the joint coordinate system convention by Grood and Suntay [13]. Maximal ROM of tibiofemoral axial rotation was thereby defined as the range between the maximal and the minimal values that occurred between implant components during the whole swing cycle.

Given clubhead velocity, 100% of the swing cycle was defined from start to end of follow‐through (start: clubhead velocity > 0.2 m/s, end: minimal overall clubhead velocity). The following timepoints of the swing cycle were additionally defined: (i) end of backswing (minimal clubhead velocity after start), (ii) impact (vertical minimum of centre of the three clubhead markers) and (iii) mid follow‐through (markers of golf shaft parallel to ground). Phases of the golf swing were chosen according to Papaliodis et al. [47].

The specific foot rotations for all subjects and trials were derived from the direction of the longitudinal axis of the front foot, measured by the vector from the lower heel (HL) marker and the marker on the second toe (TO) (Figure 1). Additionally, the clubhead and wrist velocities were determined based on the trajectory of the centre of the three markers attached to the clubhead, and, respectively, the midpoint of the two markers attached to the radial and ulnar styloid processes (Figure 1).

Ground reaction forces were measured three‐dimensionally while the players were standing on the two plates, whereby *F_y_* was in the direction of the golf swing, *F_x_* in the anterior direction of the player and *F_z_* was directed vertically upwards. Calculation of the so‐called free moment (i.e., vertical moment around the point of force application) was improved by an in situ force plate calibration method [32] and normalised to BW and body height.

### Statistics

Before statistical analysis, outcome variables were checked for normal distribution using *QQ*‐plots. Given normal distributions with minor deviations, parametric statistical analysis was carried out. To analyse the effect of bearing type and foot rotation on tibiofemoral total axial ROM during the golf swing, a linear mixed‐model analysis of variances (ANOVA) was conducted, with bearing type (MB and FB) and foot rotation (0° and 30°) as fixed effects and subject as a random effect. The null hypothesis was defined as no difference in kinematics between the different bearing groups and foot rotations. Post hoc comparisons were performed using a least significant differences approach, with a significance level of 0.05. All statistical tests were conducted using the SPSS software (SPSS v24, IBM).

To enable the temporal statistical analysis of a series of timepoints, an open‐source one‐dimensional statistical parametric mapping (SPM) code (v0.4, www.spm1d.org) was adopted, allowing the evaluation of the entire waveform of tibiofemoral axial rotation during the whole golf swing [48]. Particularly, SPMs with unpaired two‐tailed *t*‐tests were used for the comparison of the two TKA groups for all three foot rotations. To allow SPM comparisons between the two TKA designs, average tibiofemoral axial rotation during the upright standing trial of each subject was defined as the reference value.

## RESULTS

**Figure 3:**
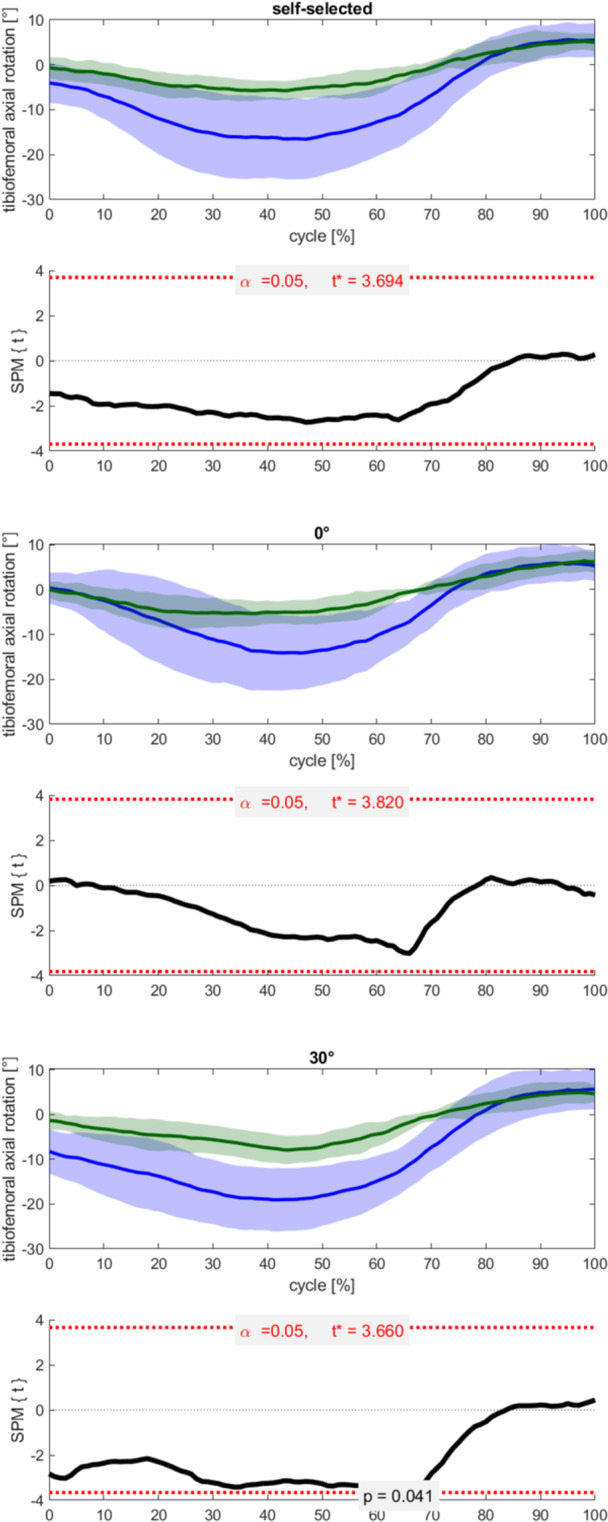
Tibiofemoral axial rotation (mean and SD, tibial relative to femoral: internal [+], external [−]) during a full golf swing cycle from start to end of follow‐through for the three foot rotations self‐selected (top row), 0° (middle row) and 30° (bottom row) for the mobile bearing (MB) (blue) and the fixed bearing (FB) (green) groups. Statistical comparison between MB and FB is based on statistical parametric mapping (SPM) with a significance level of *p* < 0.05. Zero tibiofemoral axial rotation refers to the average tibiofemoral axial rotation of the upright standing trial. SD, standard deviation

Five subjects with an MB and six subjects with an FB TKA design participated in the study (Table 1). The general rotational pattern was consistent for both implant designs, with the tibial component being maximally externally rotated relative to the femoral component around mid‐swing and maximally internally rotated at the end of follow‐through (Figure 3, Supporting Information S1: Figures S1 and S2). However, for both foot rotations (0° and 30°), the MB group exhibited a significantly larger tibiofemoral axial rotation ROM than the FB group (Table 2). Moreover, SPM analysis revealed that tibiofemoral external rotation was significantly higher in the MB group compared with the FB group for the 30° foot rotation from 63% to 67% of the swing cycle (Figure 3).

**Table 1:** Subject characteristics.

**Group**	**TKA type**	**Sex**	**Age (years)**	**Months postoperative**	**BMI (kg/m** ^**2**^ **)**	**Handicap**	**OKS**	**UCLA**
MB	Attune^TM^ CR MB	1 F, 4 M	67.2 ± 12.5	40.6 ± 16.7	25.6 ± 3.2	0–18 (*n* = 2), 19–28 (*n* = 2), > 29 (*n* = 1)	44.4 ± 4.2	8.8 ± 0.8
FB	Persona CR FB	1 F, 5 M	71.5 ± 5.8	43.5 ± 10.8	27.4 ± 0.7	0–18 (*n* = 2), 19–28 (*n* = 3), > 29 (*n* = 1)	46.8 ± 1.6	8.2 ± 0.4

*Note*: All subjects were right‐handed golfers and had the TKA on the left lead knee.

Abbreviations: BMI, body mass index; F, female; FB, fixed bearing; M, male; MB, mobile bearing; OKS, Oxford Knee Score; TKA, total knee arthroplasty; UCLA, University of California Los Angeles activity scale.

**Table 2:** Tibiofemoral axial rotation ROM—mean and SD for both groups (MB and FB) and all three foot rotations.

ROM axial rotation (°)	Self‐selected	0°	30°
MB	24.2 ± 8.1	22.8 ± 7.9* ^a,c^	26.1 ± 7.1* ^b,c^
FB	14.1 ± 4.1	14.5 ± 2.9* ^a^	15.1 ± 2.9* ^b^

*Note*: Only the 0° and the 30° foot rotations were included in the statistical analysis.

Abbreviations: FB, fixed bearing; MB, mobile bearing; ROM, range of motion; SD, standard deviation.

* Significant differences (a [*p* = 0.030], b [*p* = 0.008], c [*p *< 0.001]) based on the level of *α *< 0.05.

The 30° foot rotation led to a significantly increased ROM in tibiofemoral axial rotation compared to the 0° foot rotation in the MB group, but not the FB group (Table 2). Thereby, subjects tended to rotate the lead foot externally by a few degrees (range: 1.5°–7.2°) when instructed to stand in 0° foot rotation, while for the 30° foot rotation, an actual range of 25.2° up to 33.4° was measured based on optical motion capture. However, measured foot rotations at the start for the 0° as well as the 30° conditions were comparable between the MB and the FB groups (Table 3).

**Table 3:** Lead foot rotation at start—mean and SD for both groups (MB and FB) and all three foot rotations.

Foot rotation (°)	Self‐selected	0°	30°
MB	16.7 ± 13.7	4.1 ± 1.7	30.7 ± 2.1
FB	14.1 ± 9.0	4.4 ± 2.1	27.3 ± 3.3

Abbreviations: FB, fixed bearing; MB, mobile bearing; SD, standard deviation.

Interestingly, the chosen lead foot rotation for the self‐selected stance varied strongly between subjects, showing a range from 3.4° to 38.9°. Thereby, tibiofemoral axial rotation showed high intrasubject repeatability (reflected by the small within‐subject standard deviation [SD]), but relatively large intersubject variability in terms of motion patterns as well as ROM (Supporting Information S1: Figures S1 and S2). Hereby, the intersubject variability in tibiofemoral axial rotation was larger in the MB than the FB group, reflected by an SD in ROM of 7.9°–8.1° for the MB group in comparison to the 2.9°–4.1° for the FB group. Maximal wrist and clubhead velocities (Table 4), free moments (Table 5 and Supporting Information S1: Figure S3), as well as ground reaction forces (Supporting Information S1: Figure S4) were comparable between the MB and the FB groups for all three foot rotations.

**Table 4:** Maximal wrist and clubhead velocities—mean and SD for both groups (MB and FB) for all three foot rotations.

Max velocity (m/s)	Self‐selected	0°	30°
MB			
Wrist	4.6 ± 0.8	4.5 ± 0.6	4.5 ± 0.6
Clubhead	12.8 ± 2.8	13.1 ± 2.9	12.6 ± 3.1
FB			
Wrist	5.0 ± 0.8	5.1 ± 0.7	5.1 ± 0.8
Clubhead	13.8 ± 1.9	13.9 ± 2.1	13.9 ± 2.3

Abbreviations: FB, fixed bearing; MB, mobile bearing; SD, standard deviation.

**Table 5:** Peak free moment of the lead leg normalised to BW and HT—mean and SD for both groups (MB and FB) and all three foot rotations.

Peak free moment ×10^−3^ [BW × HT]	Self‐selected	0°	30°
MB	9.6 ± 2.4	8.1 ± 2.2	8.5 ± 2.4
FB	7.4 ± 2.1	8.1 ± 2.2	7.9 ± 1.9

Abbreviations: BW, body weight; FB, fixed bearing; HT, body height; MB, mobile bearing; SD, standard deviation.

## DISCUSSION

To our knowledge, this is the first study to provide insights into the differences in tibiofemoral kinematics during the golf swing by means of videofluoroscopy, comparing an MB with an FB TKA. Significantly greater tibiofemoral axial rotation was observed in the lead knee during the golf swing when using the MB design compared with the FB design. Externally rotating the lead foot from 0° to 30° increased tibiofemoral axial rotation in the MB design, whereas no such change was observed in the FB design.

Significantly higher magnitudes of tibiofemoral axial ROM in the lead knee during the golf swing were found for the MB compared with the FB TKA, which was independent of foot rotation at the start. Since the measured ground reaction forces and free moments were comparable between the two groups, it can be concluded that similar external loading results in higher axial rotation of the implant components for the MB compared with the FB TKA. It is assumed that the main factor leading to this finding was differences in the internal transverse plane constraints between the two TKA designs. Here, the lower constraints of the MB TKA likely allow the implant to rotate more freely in vivo, which is specifically highlighted for movement tasks that induce large transverse plane rotations, such as the golf swing. It is important to recognise that the two TKA designs investigated differ in terms of both their transverse plane constraints and the geometry of their respective femoral and tibial components. It is expected that design variability will have a greater influence on tibiofemoral axial rotation in FB designs than in MB designs, as the differences in polyethylene and femoral condyle geometry are expected to play a larger role in FB designs. Nevertheless, it is assumed that the differences in constraints introduced by mobile versus FBs will have the greatest impact on axial rotation.

Looking at the literature and different activities, inconsistent findings regarding tibiofemoral axial rotation between different TKA designs have been reported. Greater magnitudes [8, 24, 50, 65] of tibiofemoral axial rotation for MB TKA, as well as similar magnitudes [9, 35, 55, 56, 64], have been presented. In early study, greater tibiofemoral axial rotation was found during deep knee bending in 20 subjects with an MB compared against 20 subjects with an FB implant by means of videofluoroscopy, whereby the extent of axial rotation was overall smaller compared to normal knee kinematics for both TKA designs [50]. Similarly, greater magnitudes of tibiofemoral axial rotation were reported in MB than in FB designs during weight‐bearing flexion [8], during a lunge and a pivot task [24], with indications for more pronounced femoral axial rotation during more demanding tasks, such as sit‐to‐walk and turning steps in subjects with an MB versus an FB design [65]. In contrast, similar tibiofemoral axial rotation magnitudes were reported amongst major implant categories such as FB and MB in gait and deep knee bending [9], during a step up task [64], in a squatting‐to‐standing activity [35] during deep knee bending exceeding 120° flexion [56], as well as during a nonweightbearing flexion from 90° to maximal flexion [55]. However, additional rotational mobility provided by the MB design was possibly not needed during many of these investigated activities. It is therefore reasonable that challenging motion tasks that result in more extreme flexion angles and ranges of axial rotation are crucial for detecting differences in axial rotation patterns between TKA designs and seem to explain the controversy in results of previous studies. The present data support earlier findings of Zürcher et al. [66], emphasising the importance of comparing tibiofemoral axial rotation behaviour between MB and FB designs, especially for activities that induce a large range of axial rotation with below 90° flexion, such as the golf swing.

Our study results indicate that not only ROM but also the intersubject variability in tibiofemoral axial rotation was found to be higher in the MB group than the FB group. This is in agreement with a more recent study that revealed higher variability and ROM in tibiofemoral axial rotation during single‐leg lunge and pivot turns in 12 knees with an MB TKA design versus 38 knees with an FB TKA design [24]. Thereby, it was suggested that the greater variability observed in MB designs is able to more closely match healthy knee kinematics during the same activities.

In the present study, a high variability was found in the self‐selected foot rotation during the golf swing across subjects (range: 3.4°–38.9°). Therefore, the effect of foot rotation was only statistically analysed for the two controlled conditions, 0° and 30° externally rotated lead foot, whereby a change in foot rotation from 0° to 30° only significantly affected tibiofemoral axial rotation in the MB but not the FB group. The present findings indicate that a greater tibiofemoral axial rotation ROM is induced during the golf swing with a 30° externally rotated foot compared with a 0° lead foot rotation. Hereby, the 30° externally rotated lead foot rotation showed an instant effect on the tibiofemoral axial rotation at the start, as seen in the tibial external rotation of about 8° on average for the MB group. In contrast, for the FB group, external rotation of the lead foot only marginally affected tibiofemoral axial rotation at the start. Thus, it can be assumed that the higher transverse plane constraints of the FB implant limit kinematic transverse plane forward coupling of rotation from the foot to the knee. Interestingly, the 0° condition was unusual for most players and caused them to slightly rotate the foot externally despite the given instructions for 0° rotation. However, part of this difference can be explained by the fact that, for practical reasons, the 0° foot rotation condition was achieved by aligning the medial edge of the lead foot with the reference line. Foot rotation was assessed based on the angle between the longitudinal foot axis, represented by the vector connecting the heel and second toe markers. For most subjects, this will result in a difference of a few degrees.

According to a previous survey by Mallon and Callaghan [38], surgeons have not generally provided specific recommendations to patients for adapting their golfing technique following TKA. Here, the outcome of this study may provide guidance for surgeons as well as physiotherapists towards more specific recommendations for patients returning to golf after TKA. Particularly, postural changes to incorporate increased lead foot external rotation were previously shown to decrease peak external adduction moment of the lead knee following ball impact in healthy volunteers [19, 20, 37] and professional male golfers [27]. Adding to these findings, it can be concluded based on the present insights that an increase in lead foot external rotation from 0° to 30° actually increases lead knee tibiofemoral axial rotation in MB TKAs, but not in FB TKAs. Previous research has demonstrated an association between high internal tibial rotation during the golf swing and peak anterior cruciate ligament loading in ten male professional golfers, with indications from multibody dynamic musculoskeletal simulations that the subject‐specific knee joint anatomy, as well as changes in the kinematic profile of the trunk, influence soft tissue loading at the knee joint [49]. Yet, it remains unclear if a higher amount of axial rotation is desirable or not in terms of patient outcome and longevity of the implant. A larger range of axial rotation could lead to higher stresses in the surrounding soft tissues. A literature review of biomechanical analyses previously suggested that the soft tissue structures of the lead knee may be most susceptible to injury, being exposed to high strains due to high tibiofemoral axial rotation at low flexion angles (0°–30°) [1], since the anterior cruciate ligament, structures of the medial and lateral collateral ligaments, as well as the posterior medial capsule, have all been shown to be crucial in resisting axial rotation, especially in flexion angles below 30° [15, 22, 29, 39, 40, 51, 54]. As a consequence, it can be postulated that high soft tissue loading may also be a main cause for reported pain in subjects with TKA following golfing. Based on the higher axial rotation present during the swing in MB TKA patients, soft tissue loading conditions would especially be relevant for patients with an MB TKA. However, it could also be postulated that the high kinematic constraints of the FB design in combination with an activity inducing excessive tibiofemoral axial rotation may lead to higher shear stresses at the bone–implant interface which might increase the risk of implant loosening. Furthermore, since an increase in external foot rotation was found to additionally increase tibiofemoral axial rotation, one could draw the conclusion that golfers with an FB TKA should not further externally rotate the lead foot but rather use a foot rotation towards 0°. However, it should also be considered that modifications to a golfer's stance could have negative implications on comfort, performance and whole‐body kinematics that need further investigation. Future computational modelling studies, optimally based on in vivo assessed kinematic and kinetic data, should aim to assess the influence of TKA design as well as lead foot rotation on the loading of the surrounding ligaments as well as the shear stresses at the bone–implant interface.

The tibiofemoral kinematic assessment by means of videofluoroscopy performed in this study was only possible using a modified golf club, which was shorter than a regular club, as it would not be possible to perform a full swing within the C‐arm setup using a regular club. In addition, it was also not possible to hit a ball. However, maximal wrist velocities, known to be a significant predictor of club‐head velocity [57], were comparable to swings performed with a regular seven‐iron golf club (Supporting Information S1: Table S1). Moreover, the setup allowed the assessment of accurate tibiofemoral implant kinematics without errors due to soft tissue artefacts. The small number of subjects included in our study limits its statistical power. Furthermore, as the study did not have a randomised, blinded design, other influencing factors such as preoperative ROM, leg alignment, ligament condition and level of activity were not controlled for.

## CONCLUSION

Given the specific motion pattern of the knee joint during golfing, the question remains whether component type matters in golfers after TKA. The present results suggest that the MB design allows more tibiofemoral axial rotation than the FB design when performing a golf swing. Furthermore, the present data showed that a change in foot rotation from a 0° to a 30° externally rotated lead foot increases tibiofemoral axial rotation of the MB design but not the FB design. However, it remains unclear if the higher rotation is preferential in terms of longevity and soft tissue pain. Further computational modelling based on in vivo kinematic and kinetic data is recommended to evaluate how this change in rotation affects ligament strain as well as shear stresses at the bone–implant interface. Knowledge of the influence of bearing type and lead foot rotation on TKA kinematics and loading is expected to guide implant selection and recommendations for foot positioning during the golf swing for patients who intend to play golf again after TKA. The present data will serve as input data for musculoskeletal and finite element modelling to improve the understanding of occurrences of impairments such as loosening or soft tissue pain in TKA patients playing golf.

## AUTHOR CONTRIBUTIONS

**Renate List:** Conceptualisation; data curation; formal analysis; investigation; methodology; project administration; resources; software; supervision; validation; visualisation; writing—original draft, writing—review and editing. **Katja Oberhofer:** Conceptualisation; writing—original draft; writing—review and editing. **Nils Horn:** Conceptualisation; investigation; resources; writing—review and editing. **Samara Monn:** Data curation; formal analysis; investigation; project administration; software; visualisation; writing—review and editing. **Danielle Hinny:** Data curation; formal analysis; investigation; software; writing—review and editing. **Philipp Bänteli:** Data curation; formal analysis; investigation; software; writing—review and editing. **Kevin Wunderlin:** Data curation; formal analysis; investigation; software; writing—review and editing. **William R. Taylor:** Conceptualisation; resources; writing—review and editing. **Stephen J. Ferguson:** Conceptualisation; resources; supervision; writing—review and editing. **Tomas Drobny:** Conceptualisation; writing—review and editing. **Stefan Preiss:** Conceptualisation; resources; writing—review and editing. **Pascal Schütz:** Conceptualisation; data curation; formal analysis; investigation; methodology; project administration; resources; software; supervision; validation; writing—review and editing.

## CONFLICT OF INTEREST STATEMENT

The authors declare no conflicts of interest.

## ETHICS STATEMENT

The study was performed in line with the principles of the Declaration of Helsinki. Approval was granted by the local ethics committee of the canton of Zurich (BASEC‐Nr. 2018‐01763). All subjects provided written informed consent prior to study participation in accordance with local ethical regulations.

## Supporting information

supporting information.

## Data Availability

The data that support the findings of this study are available on request from the corresponding author. The data are not publicly available due to privacy or ethical restrictions.
