# Supplementary material for: Tibiofemoral axial rotation during the golf swing is influenced by total knee arthroplasty bearing type and foot rotation
Source: J Exp Orthop. 2025 Dec 10;12(4):e70529. doi: 10.1002/jeo2.70529 (PMC12690408; doi:10.1002/jeo2.70529)
Supplement: Supplementary file 1 — supporting information. [file JEO2-12-e70529-s001.pdf]

Supplementary Material

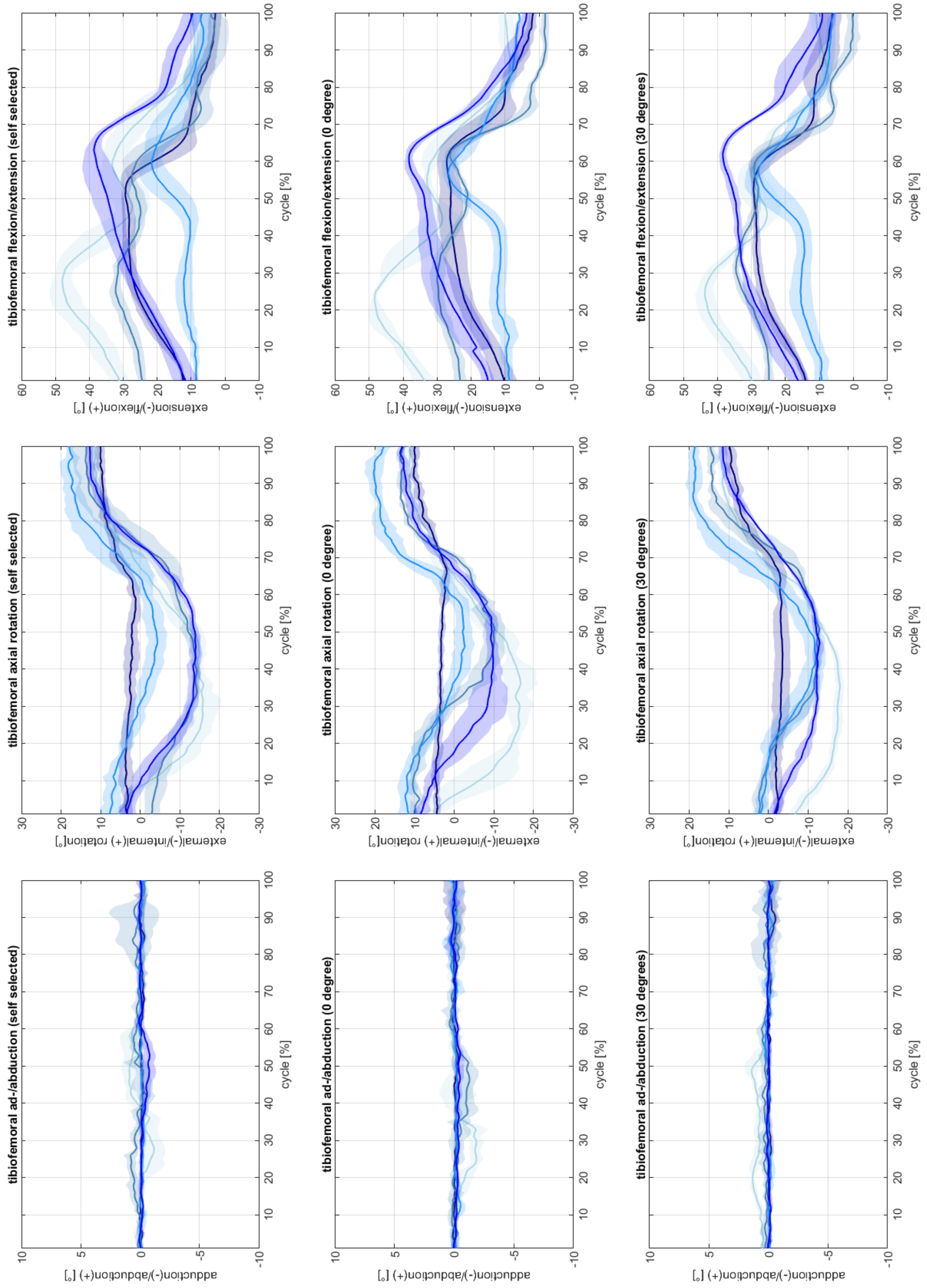

Fig. S1 Subject means (thick line) and SD (shaded area) of tibiofemoral joint rotations (frontal, transverse and sagittal plane) for the five mobile bearing (MB) patients throughout full cycles of the golf swing

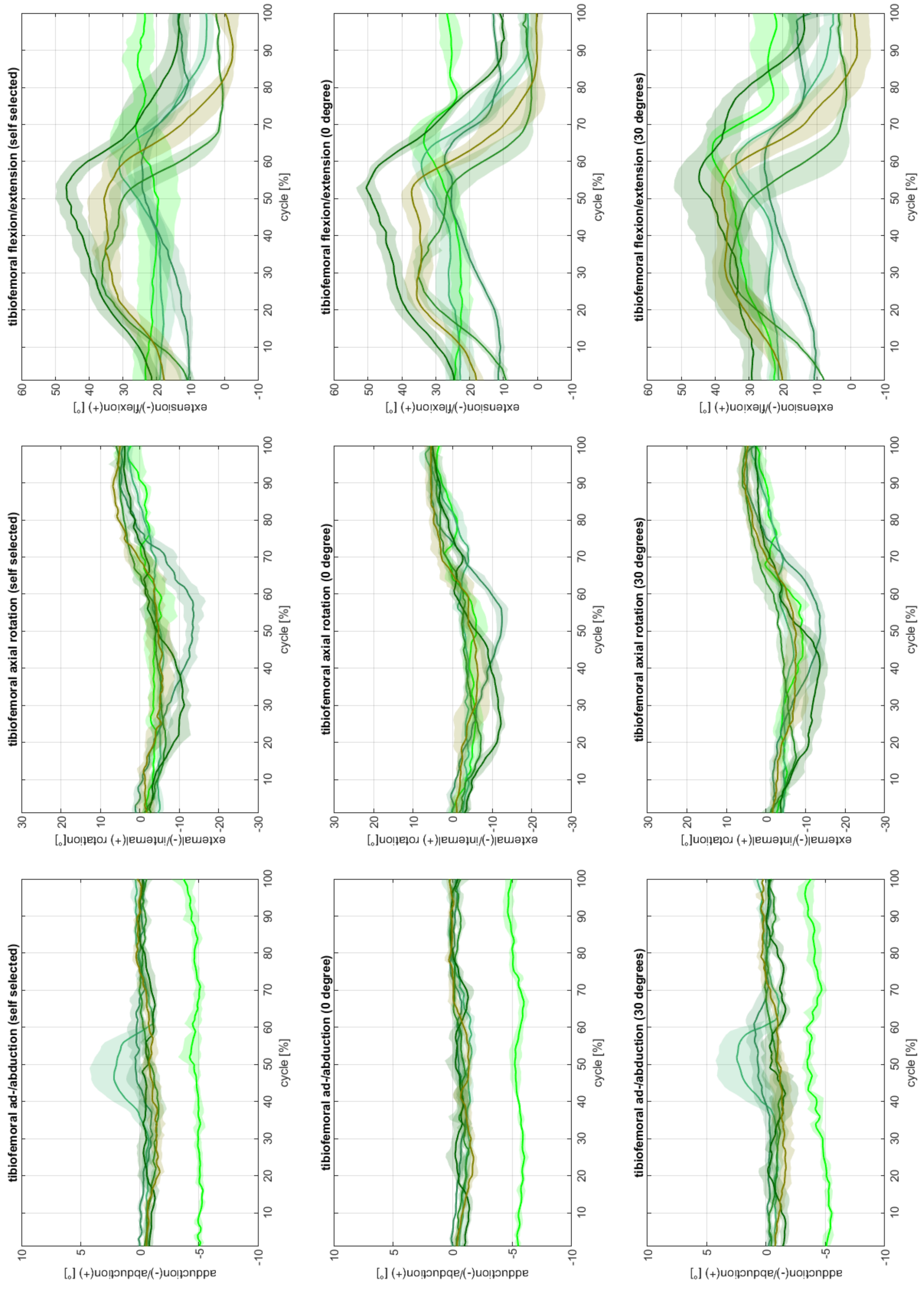

Fig. S2 Subject means (thick line) and SD (shaded area) of tibiofemoral joint rotations (frontal, transverse and sagittal plane) for the six fixed bearing (FB) patients throughout full cycles of the golf swing

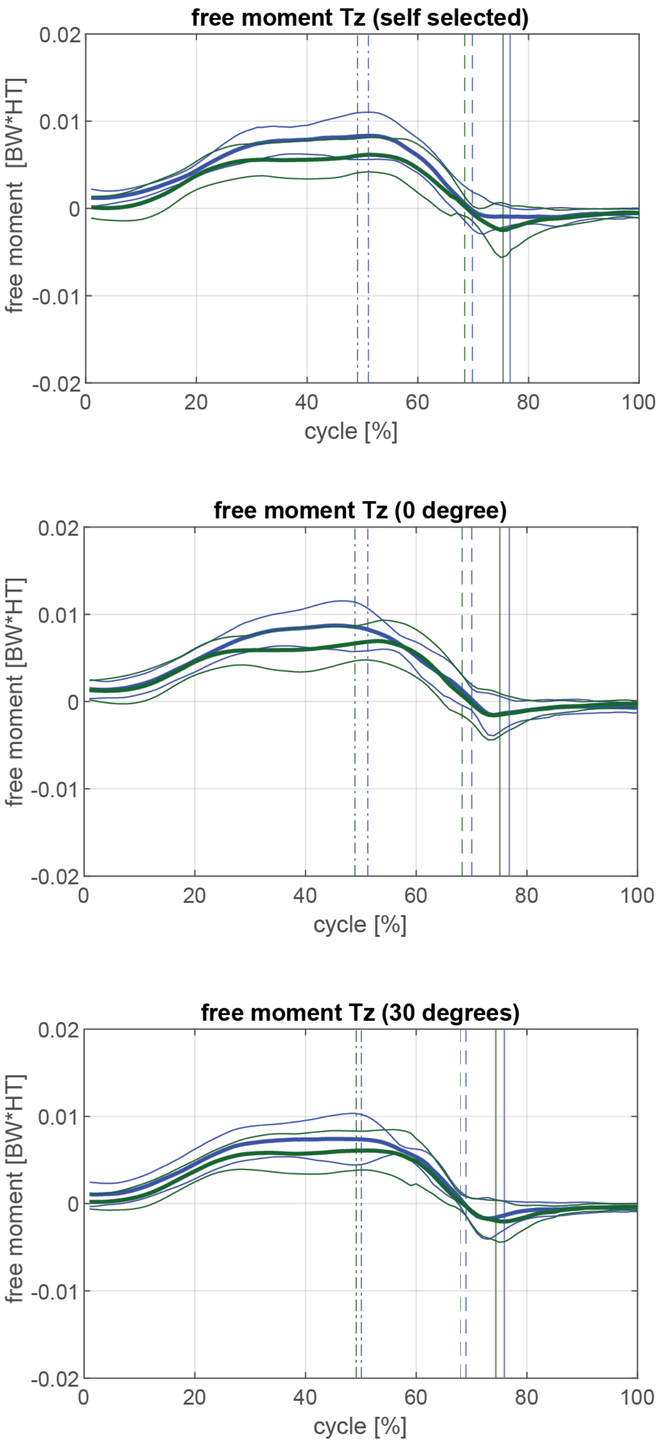

Fig. S3 Free moment of the lead leg normalized to body weight (BW) and body height (HT) (mean and SD) during a full golf swing cycle from start to end of follow-through for the three foot rotations self-selected (top row), 0° (mid row) and 30° (bottom row) for the mobile bearing (MB) (blue) and the fixed bearing (FB) (green) groups. The vertical lines represent the group means of the events end of backswing (dash dotted), impact (dashed) and mid follow-through (full line).

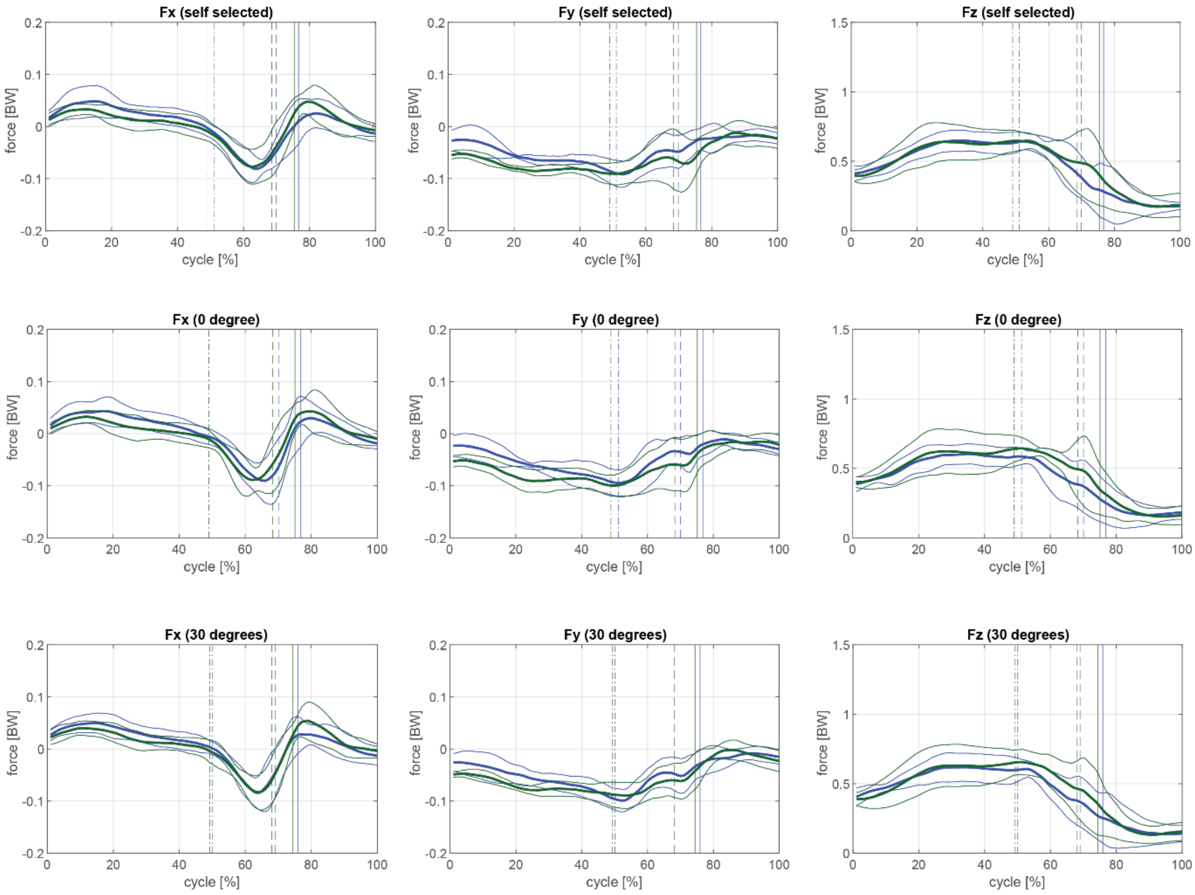

Fig. S4 Ground reaction forces of the lead leg normalized to body weight (BW) (mean and SD) in antero-posterior (left column), medio-lateral (mid column) and vertical (right column) direction during a full golf swing cycle from start to end of follow-through for the three foot rotations self-selected (top row), 0° (mid row) and 30° (bottom row) for the mobile bearing (MB) (blue) and the fixed bearing (FB) (green) groups. The vertical lines represent the group means of the events end of backswing (dash-dotted), impact (dashed) and mid follow-through (solid line).

Table S1. *Maximal wrist velocities during the golf swing with a regular 7-iron club – mean and SD for both groups (mobile (MB) and fixed bearing (FB)) and all three foot rotations.*

Max Velocity [m/s]		self-selected	0°	30°
MB	wrist	4.6 ± 0.7	4.4 ± 0.5	4.5 ± 0.0
FB	wrist	4.8 ± 0.6	4.5 ± 0.5	4.7 ± 0.7
